# Pedestrian Flow Prediction and Route Recommendation with Business Events †

**DOI:** 10.3390/s22197478

**Published:** 2022-10-02

**Authors:** Jiqing Gu, Chao Song, Zheng Ren, Li Lu, Wenjun Jiang, Ming Liu

**Affiliations:** 1 School of Computer Science and Engineering, University of Electronic Science and Technology of China, Qingshuihe Campus, Chengdu 611731, China; 2 College of Computer Science and Electronic Engineering, Hunan University, Changsha 410082, China

**Keywords:** matrix factorization, pedestrian flow prediction, route recommendation, embedding learning

## Abstract

Due to the potential economic benefits, pedestrian flow is considered an essential indication of public spaces. Pedestrian flow prediction is designed to assist operators in making decisions (such as shopping center owners). Operators hold certain events, such as sales promotions, to attract surrounding pedestrians; we refer to this type of event as a business event. Business events attract pedestrian flows, which means business opportunities for the merchants. Moreover, their placement will affect the distributions of the pedestrian flows. However, deciding which route is chosen for a specified event is difficult. To the best of our knowledge, we are the first to consider business events when predicting pedestrian flow. In this paper, we investigate two problems: one is pedestrian flow prediction with business events, and the other is route recommendation for business events. First, we propose an Attraction-Based Matrix Factorization model (ABMF) to efficiently predict the pedestrian flow with business events, which introduces the attraction index of different categories to pedestrians in matrix factorization. Second, we leverage the Skip-gram mode to learn the latent representations and improve the pair-wise ranking loss to a flow-aware-based method (SG-FWARP), which aims to learn events’ latent representations for route recommendation. Compared with other state-of-the-art methods, the experimental results show ABMF can predict pedestrian flow matrix with a similarity of over 0.9 compared with the ground truth, and SG-FWARP can recommend routes for business events with high accuracy.

## 1. Introduction

The rapid progress of urbanization brings prosperity to businesses. A large number of human beings visit the business district, which forms dynamic pedestrian flows between Points of interests (POIs). Mobile crowdsourcing data from location-based social network services (LBSNs) provide information on individuals’ preferences for routes and locations [1,2,3]. Pedestrian flow prediction provides crucial information to operators for decision-making. For instance, by utilizing crowdsourced trajectories, pedestrian flow analysis enhances the structure of technological show events [4], and pedestrian flow prediction in vast road networks is used to benefit the German outdoor advertising market [5]. The pedestrian flow provides the operators purchasing power and has the potential ability to benefit them. Additionally, the operators frequently organize events to draw the nearby pedestrian flow in order to gain greater commercial benefits. Researchers studied the prediction with different types of events in real life. For example, the work [6] predicts the crowdedness of POIs in order to enhance personalized trip recommendations by avoiding crowds. The work [7] forecasts attendance of the activity in event-based social networks (e.g., Douban).

**Figure 1:**
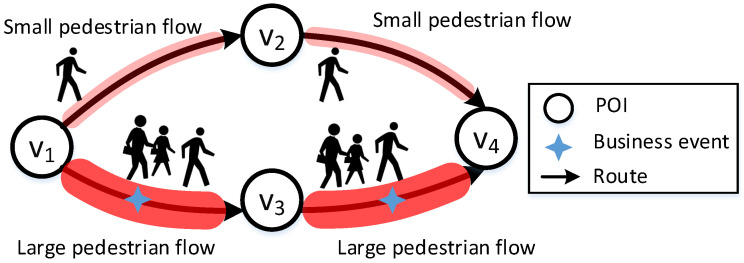
Business event increases the pedestrian flow of a route.

However, events that occur along the route are rarely studied by academics, such as sales promotions, and we refer to this novel type of event as *business events*. Unlike previous studies on pedestrian flows, we focus on pedestrian flow prediction under business events. Business events are frequently hosted in areas with high pedestrian flows because event organizers want to draw attention to their products by gathering pedestrian flow. Figure 1 depicts the effect of a business event on pedestrian flow: there are two paths from $v_{1}$ to $v_{4}$, both of which pass through $v_{2}$ and $v_{3}$. Two business events are associated with the two routes $v_{1}\to v_{3}$ and $v_{3}\to v_{4}$, respectively. These events attract a greater number of pedestrians. As business events are scheduled to be placed, predicting pedestrian flow with business events is a big challenge. The challenge is that different business events on different routes will result in varied distributions of the overall pedestrian flow.

In this paper, by utilizing mobile crowdsourcing data, we study how business events affect pedestrian flows. Based on historical pedestrian flows, we present an Attraction- Based Matrix Factorization model (ABMF) to predict pedestrian flows with business events accurately, which introduces the attraction index of different categories to pedestrians in matrix factorization. In some cases, the operator only knows which event he plans to hold but does not know which route fits the event. Thus, it is essential to recommend routes for a business event. So, we investigate which routes fit for holding an event, and recommend top-*N* routes for a specified business event. The goal of top-*N* route recommendation is to recommend for each event *N* routes that are most appropriate for it. To achieve this goal, by leveraging the Skip-gram model [8], we learn the latent representations for a route to capture the contextual information in sequences. By considering the flow factors, we improve the pair-wise ranking loss to a flow-aware-based method, which aims to learn events’ latent representations for route recommendation. The main contributions of this paper are:

1. As far as we know, we are the first to take business events into account when predicting pedestrian flow.
2. To predict pedestrian flow based on business events, we present the ABMF model, and its highlight lies in that it introduces the attraction index of different categories to pedestrians in matrix factorization.
3. To recommend routes for event placement, we learn route representations based on the Skip-gram model and consider the flow factors to recommend top-*N* routes for events.
4. We compare the performance of the proposed two methods with state-of-the-art solutions on a simulation dataset and real-world datasets, and experiment results reveal that our algorithms outperform the baselines.

This paper is organized as follows: Section 2 reviews related work. Section 3 shows the data model and problem definition. We detail the ABMF method and the parameter estimation in Section 4. Then, we introduce the extension of route recommendation for events in Section 5. We report the experimental results in Section 6 and conclude our paper in Section 7.

## 2. Related Work

In this section, we review the related work from the following aspects:

(1) Pedestrian Flow Analysis: Pedestrian flow analysis, which is used to extract information from stored trajectories via pedestrian flow modeling, simulation, and optimization, has garnered increased attention. In the literature on pedestrian modeling, a number of modeling techniques have already been proposed at microscopic and macroscopic scales, including trajectory clustering for extracting similar trajectories from a dataset [9]. An analysis of pedestrian flow in complicated scenes computes a representation of the main pedestrian flows [10]. The work [11] predicts the influence of urban environment layout on the spatial distribution of pedestrian flows. Based on the pedestrian trajectories, the work [4] shows a technique for real pedestrian track analysis during an actual exhibition. There are numerous approaches for simulating pedestrian traffic. The work [12] simulates the pedestrian flow by considering the interaction among pedestrians. Kaminka et al. [13] discuss a model of urban pedestrian flow based on agents.

(2) Pedestrian Flow Prediction: Predicting pedestrian flow is a fundamental topic in urban computing [14]. Previous research focuses on predicting people’s movements using their past location data [15,16]. In recent years, researchers have developed methods to forecast the city’s crowd flow in a variety of situations, including taxi and bike movements [17]. Zhou et al. [18] propose MOHER to predict the potential crowd flow in a certain mode, which uses the LTSM module to predict the sequential flow. To predict the crowd flow over the entire city, Zhang et al. [19] develop an end-to-end ST-ResNet structure based on unique spatio-temporal data features. Some studies concentrate on predicting pedestrian flow: Eravci et al. [20] forecast the scale of pedestrian flows to provide commercial advice; Ma et al. [21] propose an approach that automatically predicts crowd density in the short term, which presents a prediction algorithm using v-support vector regression (vSVR). Duan et al. [22] introduce a complementary attention gated network to predict pedestrian trajectory, which captures both frequent and peculiar modals in spatial and temporal patterns.

(3) Urban Computing and Business Recommendation: The rich human mobility data generated in urban spaces reflects a city’s underlying problems, helping urban planners to formulate plans effectively. Big data-driven urban computing is a popular field with many valuable applications. Li et al. [23] select locations for ambulance stations. Bao et al. [24] plan bike lanes relaying on the sharing-bikes’ trajectories. Liu et al. [25] aim to select billboard locations based on the large-scale taxi trajectories. The recommendation technique is employed in business category selection, store location selection, etc. Zhao et al. [26] utilize the data from location-based social networks to recommend new business categories in a partitioned business district, which mines the business opportunities and guides the planners to open new commercial shops in certain categories in a specific district. Some researchers employ the business data to support the business owners in LBSNs, designing the zone recommendation system [27], business prediction system [28], and retail allocation system [29]. Lu et al. [30] apply user and business properties for personalized business recommendation.

(4) Sequential Modeling and Embedding Learning: Modeling the sequential pattern is an important technique in the recommendation field. Many works apply the Markov chain in the successive check-ins to observe the sequential pattern. The studies in [31] learn the transitive pattern of categories in sequential check-ins. Zhang et al. [32] learn the transitive probability from the additive Markov chain to recommend POIs. With the success of deep learning, the neural network has been utilized to model the check-in sequences. The work [33] applies the word2vec framework to model the check-in sequences to capture the sequential contexts. The prior works [31,33] inspire us to capture the sequential pattern from user travel trajectories to improve business event recommendation. The word2vec framework [8] is a neural language model to learn latent representations of words effectively. The main idea is to observe a word’s contextual relations in sentences, which perform better than the viewpoint of word similarity and transitivity in sentences. With the success of the framework in capturing the contextual correlations of items, the word2vec framework is widely used for user modeling [34] and POI modeling [35], etc. For example, Liu et al. [33] adopt a Skip-Gram model in POI recommendation by capturing the sequential POI check-ins and performs better than the Markov chain model. However, the work [33,36] neglects the crowd flow influence, so we propose a flow-aware model to recommend routes for the business event. Compared to these studies, the main difference lies in our proposed methods naturally focusing on predicting the pedestrian flow with business events and recommending routes for business events. This prediction assists operators in making decisions on where to place business events, increasing possible economic gains.

## 3. Preliminaries

The motivation to predict pedestrian flow with business events is as follows: The business events that are held along the route will attract pedestrian flow, which could have advantages and is the driving force behind the prediction of pedestrian flow with business events. This section explains the data model and defines the problem of predicting pedestrian flow under business activities. The most common notations used in this paper are listed in Table 1.

### 3.1. Data Model

Assume there are *n* locations (or POIs) and *k* business events in a region, which is represented by a fully connected graph G=(V,E), where *V* represents the set of locations and *E* represents the set of routes. We assume that, at most, one business event will occur on the route between two locations. We treat several business events that take place between two locations as a single event with a mixed preference, which covers the preference of every business event on this route. We describe a pedestrian flow matrix as follows:

**Definition** **1.** *A pedestrian flow matrix is a n-order matrix denoted as M, with the element $m_{i}j$ referring to the pedestrian flow between locations $v_{i}$ and $v_{j}$ under a set of business events.*

The pedestrian flow history dataset is defined as MA, $MA=\{\left(M^{1},A^{1},R^{1}\right),\left(M^{2},A^{2},R^{2}\right),\left(M^{i},A^{i},R^{i}\right),\ldots \}$, where $M^{i}$, $A^{i}$ and $R^{i}$ denote the pedestrian flow matrix, the set of business events and the position matrix of each business event during the *i*th time window (i∈[1,n]), respectively. Here, $R^{i}$ and $M^{i}$ are of equal size. The attraction of business events in $A^{i}$ and their interactions influence the pedestrian flow matrix $M^{i}$. It should be noted that all MA data is collected either during the week or on the weekends (holiday).

### 3.2. Problem Definition

**Figure 2:**
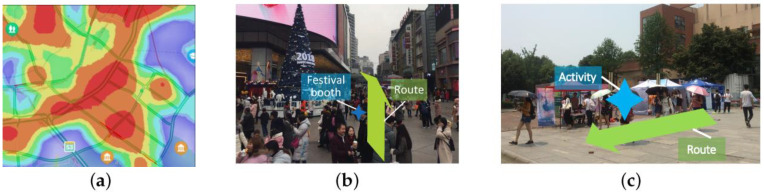
An example of a business event: (**a**) Heat map of crowds; (**b**) Festival booth; (**c**) Business event in campus.

Figure 2a depicts a heat map of crowds for a district. The business event, i.e., a festival booth in Figure 2b, is taken on the route (The terms “route” and “edge” are used interchangeably in this paper.), and the business event in Figure 2c is taken on a route between the POIs in the campus. The green arrows in Figure 2b,c reflect the pedestrian flow on the original route, while the blue signs represent the event on the route. The problem of pedestrian flow prediction with business events is defined as follows:

**Problem** **1** (Pedestrian flow prediction with business events.)**.** *Given the historical data MA with (s−1) time windows, the set of business events $A^{s}$ at the $s^{th}$ time window, and the position matrix $R^{s}$ of $A^{s}$, we aim to predict the pedestrian flow matrix $M^{s}$ at the $s^{th}$ time window.*

Business events have an impact on the pedestrian flow matrix. Typically, business events attract pedestrians to visit. Figure 2b shows how visitors are drawn to the events. The phenomenon demonstrates that business events can attract the pedestrian flow for the routes they place. As a result, pedestrian traffic modeling will help operators make decisions about where to arrange business events. The challenge is that different business events placed on different routes result in varying distributions of the overall pedestrian flow.

## 4. Predicting Pedestrian Flow

**Figure 3:**
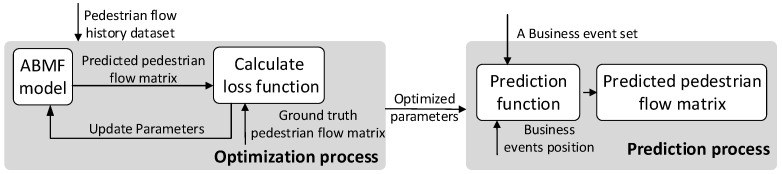
Framework of pedestrian flow prediction with business events.

This section presents an Attraction-Based Matrix Factorization (ABMF) model to overcome the aforementioned challenge. Figure 3 outlines the structure of pedestrian flow prediction with business events, including the optimization and prediction procedures.

### 4.1. ABMF Model

Unlike the traditional matrix factorization [37], we construct a location–location matrix, where each $m_{ij}$ represents the pedestrian flow between location $v_{i}$ to location $v_{j}$. Let $X\in R^{z\times n}$ and $Y\in R^{z\times n}$ be the latent location feature matrix and the latent factor feature matrix [38], with column vectors $X_{i}$ and $Y_{i}$ representing the *z*-dimensional location-specific and factor-specific feature vectors of location $v_{i}$. In particular, the vector of location feature represents the location’s preference for corresponding properties, and the vector of factor feature captures the properties level of location $v_{i}$, where each property level indicates the ability to attract visitors. For instance, $X\in R^{3\times 2}$ and $Y\in R^{3\times 2}$, $X_{1}={\left(0.8,0.6,0.1\right)}^{T}$, $Y_{1}={\left(200,60,30\right)}^{T}$. The elements in $X_{1}$ represent the preference for three categories of location 1, which correspond to the degree of belonging to a category. The elements in $Y_{1}$ represent the ability to attract visitors for corresponding categories of location 1. Therefore, the location 1 can attract 0.8×200+0.6×60+0.1×30=199 visitors. Similarly, we also construct two matrices for business events. We denote $H\in R^{z\times |C|}$ and $F\in R^{z\times |C|}$ as the latent business event feature matrix and the latent factor feature matrix of business event, where the column vectors $H_{j}$ and $F_{j}$ represent the *z*-dimensional business event-specific and factor-specific feature vectors of business event $a_{j}$. *C* denotes a set of categories $C=\{c_{1},c_{2},\cdots ,c_{\left|C\right|}\}$ and |C| is the amount of categories of business events. The business event feature vector, in particular, reflects the preference of the business event for corresponding properties, whereas the factor feature vector captures the property level of the business event $a_{i}$, which is the attractive crowd index for each property. We create an undirected graph G=(V,E), where $V=\{v_{1},v_{2},\cdots ,v_{\left|V\right|}\}$ signifies the set of the locations and $e_{ij}$ represents the edge (route) between $v_{i}$ and $v_{j}$. Let $A=\{a_{1},a_{2},\cdots ,a_{\left|A\right|}\}$ denote the set of all the business events. If the visitors are visiting at $v_{i}$, and then they are attracted to visit $v_{j}$, the pedestrian flow from $v_{i}$ to $v_{j}$ is formed. When a business event $a_{k}$ ($a_{k}\in A$) takes place on $e_{ij}$, the pedestrian flow on $e_{ij}$ includes four aspects: (1) the pedestrian flow from $v_{j}$ to $v_{i}$; (2) the pedestrian flow from $v_{i}$ to $v_{j}$; (3) the pedestrian flow that $a_{k}$ attracts from all the locations; (4) the pedestrian flow that $a_{k}$ attracts from other business events, excluding the pedestrian flow that other business events attract from $a_{k}$. As a result, we integrate X,Y,H,F together to model the pedestrian flow between $v_{i}$ and $v_{j}$.

**Figure 4:**
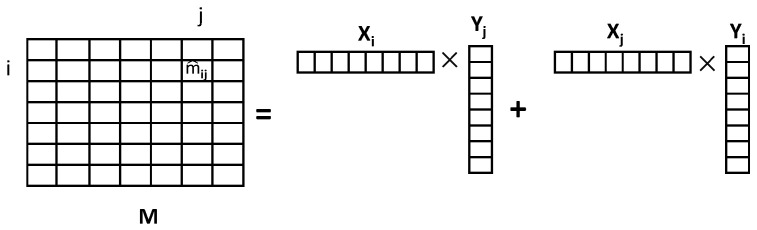
Vector representations for pedestrian flow matrix *M*.

The current set of a business event is denoted as $A^{\prime }$, where $A^{\prime }\subset A$. The predicted pedestrian flow ${\hat{m}}_{ij}$ between location $v_{i}$ and location $v_{j}$ is defined as follows:

(1) $$\begin{array}{cc} {\hat{m}}_{ij} & =X_{i}^{T}Y_{j}+X_{j}^{T}Y_{i}+I_{ij}^{a_{k}}\sum_{s\in V}\frac{1}{\left|V\right|}X_{s}^{T}F_{f(a_{k})}+I_{ij}^{a_{k}}(\sum_{a_{m}\in A^{\prime }/a_{k}}\frac{1}{|A^{\prime }/a_{k}|}H_{f(a_{m})}^{T}F_{f(a_{k})}\\ & -\sum_{a_{m}\in A^{\prime }/a_{k}}\frac{1}{|A^{\prime }/a_{m}|}H_{f(a_{k})}^{T}F_{f(a_{m})}) \end{array}$$

where the function f(ak) converts ak into its category index. The indicator function, $I_{ij}^{a_{k}}$, is equal to 1 if the $a_{k}$ takes place on $e_{ij}$ and to 0 otherwise. $A^{\prime }/a_{k}$ represents the business events set excluding $a_{k}$, while $A^{\prime }/a_{m}$ represents the set of business events excluding $a_{m}$. $\frac{1}{V}$ is the attraction influence probability of $a_{k}$ across all POIs. The attraction influence probability of $a_{k}$ on other business events is $\frac{1}{|A^{\prime }/a_{k}|}$, while the attraction influence probability of other business events on $a_{k}$ is $\frac{1}{|A^{\prime }/a_{m}|}$. Note that $m_{ii}=0$(i∈[1,n]). If there is no event between locations $v_{i}$ and location $v_{j}$, $m_{ij}$ is represented as: ${\hat{m}}_{ij}=X_{i}^{T}Y_{j}+X_{j}^{T}Y_{i}$, as shown in Figure 4.

Inspired by [38], the elements in the pedestrian flow matrix are supposed to be drawn from a Gaussian distribution with the mean as stated in Equation (1) and the precision as $\lambda_{m}$. We apply zero-mean spherical Gaussian priors on location and factor feature vectors with precisions of $\lambda_{x}$ and $\lambda_{y}$, respectively. Similarly, we apply zero-mean spherical Gaussian priors with the precision of $\lambda_{h}$ and $\lambda_{f}$ to business event and factor feature vectors. Therefore, using a Maximum-a-Posteriori (MAP) estimation, we derive the objective function for *X*, *Y*, *H*, and *F* in Equation (2), where ${\left\|\cdot \right\|}_{F}$ signifies the Frobenius norm to avoid overfitting.

When ${\hat{m}}_{ij}$ is substituted for Equation (1), the ABMF model is learned by the following objective function, where ${\hat{m}}_{ij}$ is the estimated value as indicated in Equation (1).

(2) $$\begin{array}{cc} Loss & \;=\;\min_{X,Y,H,F}\frac{\lambda_{m}}{2}\sum_{i=1}^{n}\sum_{j=1}^{n}{\left(m_{ij}-{\hat{m}}_{ij}\right)}^{2}\;+\;\frac{\lambda_{x}}{2}{\left\|X\right\|}_{F}^{2}+\;\frac{\lambda_{y}}{2}{\left\|Y\right\|}_{F}^{2}\;+\;\frac{\lambda_{h}}{2}{\left\|H\right\|}_{F}^{2}\;+\;\frac{\lambda_{f}}{2}{\left\|F\right\|}_{F}^{2} \end{array}$$

### 4.2. Parameter Estimation

We utilize stochastic gradient descent (SGD) to learn the objective function. For each training instance, we update the associated parameters as well as the gradient’s ascending direction to learn the model. The gradients of Loss with regard to the latent factor features are as follows:

(3) $$\begin{array}{ccc} \; & \frac{\partial Loss}{\partial X_{i}}\;=\;\lambda_{m}\sum_{j=1}^{n}\left(m_{ij}\;-\;{\hat{m}}_{ij}\right)\left(-Y_{j}\;-\;Y_{i}\right)\;+\;I_{ij}^{a_{k}}\frac{F_{f(a_{k})}}{\left|V\right|}\;+\;\lambda_{x}X_{i} & \end{array}$$

(4) $$\begin{array}{ccc} \; & \frac{\partial Loss}{\partial Y_{j}}\;=\;\lambda_{m}\sum_{i=1}^{n}\left(m_{ij}-{\hat{m}}_{ij}\right)\left(-X_{i}\;-\;X_{j}\right)\;+\;\lambda_{y}Y_{j} & \end{array}$$

(5) $$\begin{array}{ccc} \; & \frac{\partial Loss}{\partial H_{c}}\;=\;\lambda_{m}\sum_{i=1}^{n}\sum_{j=1}^{n}\left(m_{ij}-{\hat{m}}_{ij}\right)I_{ij}^{a_{k}}\tilde{F}+\lambda_{h}H_{c} & \end{array}$$

(6) $$\begin{array}{ccc} \; & \frac{\partial Loss}{\partial F_{r}}\;=\;\lambda_{m}\sum_{i=1}^{n}\sum_{j=1}^{n}\left(m_{ij}-{\hat{m}}_{ij}\right)I_{ij}^{a_{k}}\tilde{H}+\lambda_{f}F_{r} & \end{array}$$

We define the following variables to simplify the formula:

(7) $$\begin{array}{ccc} \; & \tilde{F}=\frac{|D_{1}|}{|A^{\prime }/a_{k}|}F_{f(a_{k})}-\sum_{a_{m}\in A^{\prime }/a_{k}}\frac{1}{|A^{\prime }/a_{m}|}F_{f(a_{m})} & \end{array}$$

(8) $$\begin{array}{cc} \; & \tilde{H}\;=\;\frac{1}{\left|V\right|}\sum_{s=1}^{\left|V\right|}X_{s}+\sum_{a_{m}\in A^{\prime }/a_{k}}\frac{1}{|A^{\prime }/a_{k}|}H_{f(a_{m})}\;-\;\frac{|D_{2}|}{|A^{\prime }|-1}H_{f(a_{k})} \end{array}$$

where $D_{1}$ is the business events set where $a_{m}\in D_{1}$ and satisfies $a_{m}\in A^{\prime }/a_{k}\&f\left(a_{m}\right)=c$; $D_{2}$ is the business events set where $a_{m}\in D_{2}$ and satisfies $a_{m}\in A^{\prime }/a_{k}\&f\left(a_{m}\right)=r$.

The following are the details of Algorithm 1: the inputs consist of the abstracted graph G=(V,E), the historical data MA, the set of all the business events *A*, regularization coefficients, the dimension *z*, business events category number |C| and the learning rate η. In lines 1–7, for each pair of historical data in MA, it randomly initializes X,Y,H,F. Then, each column vector of X,Y,H, and F is simultaneously updated as follows based on the gradients and step size η:

(9) $$\begin{array}{cc} \; & X_{i}\leftarrow X_{i}-\eta \frac{\partial Loss}{\partial X_{i}} \end{array}$$

(10) $$\begin{array}{cc} \; & Y_{j}\leftarrow Y_{j}-\eta \frac{\partial Loss}{\partial Y_{j}} \end{array}$$

(11) $$\begin{array}{cc} \; & H_{c}\leftarrow H_{c}-\eta \frac{\partial Loss}{\partial H_{c}} \end{array}$$

(12) $$\begin{array}{cc} \; & F_{r}\leftarrow F_{r}-\eta \frac{\partial Loss}{\partial F_{r}} \end{array}$$

After that, the algorithm computes the learned matrices $X^{i},Y^{i},H^{i},F^{i}$ for each pair of $\left(M^{i},A^{i}\right)$. In lines 8–12, after training all the samples, for each $X_{ab}^{i}\left(i\in \left[1,n\right]\right)$, the mean value is calculated excluding the maximal $X_{ab}^{i}$ and the minimum $X_{ab}^{i}$, because we want to lessen the impact of the maximum and the minimum on the mean value. Then, we obtain the final optimized *X* and apply the same method to calculate Y,H,F.
**Algorithm 1:** The ABMF Optimization
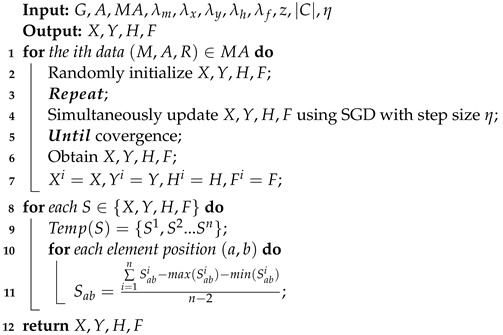


When making predictions, the operator plans to allocate a set of business events $A_{s}$ in the region with the position matrix $R^{s}$. Based on the learnt matrices, we use Equation (1) to predict each element of the pedestrian flow matrix.

## 5. Extension: Route Recommendation for Business Events

In this section, we make an extension of the pedestrian flow prediction with business events. In Problem 1, we define the problem of predicting pedestrian flow under a set of business events whose position is known in advance. In some cases, the operator only knows which event he plans to hold but doesnot know which route fits the event. Thus, it is essential to recommend routes for a business event. This is why we make the extension.

In the following subsections, we first introduce the concepts and problems. In order to recommend routes for business event and attract more pedestrian flows, we then apply a Skip-gram-based model to represent each route like representing word vector in Section 5.2. Finally, we improve the pair-wise ranking loss to a flow-aware-based method by considering the flow factors in Section 5.3, which aims to learn events’ latent representations for top-*N* route recommendation by minimizing the F-WARP loss.

### 5.1. Concepts and Problem Definition

The set of business events is denoted by $A=\{a_{1},a_{2},...\}$. The set of all routes in a travel graph is denoted by $E=\{e_{1},e_{2},...\}$. The travel trajectories of users are defined as follows:

**Definition** **2** (Travel trajectories)**.** *The set of travel trajectories is denoted by $S=\{S_{1},S_{2},...\}$, where $S_{i}$ refers to the ith travel trajectory and $S_{i}=\left\{e_{1},e_{2},...,e_{i},...\right\}$, $e_{i}$ denotes a route in a travel trajectory. Note that $S_{i}$ is the subset of E.*

**Definition** **3** (Target route and context route)**.** *In a travel trajectory $S_{u}$, the chosen $e_{i}$ is the target route, and other routes in $S_{u}$ are context routes.*

**Problem** **2** (Routes recommendation for an event)**.** *Given a business event a and users’ travel trajectories set E, the task is to recommend routes to hold this event, which aims to attract more people to visit this event so as to bring more benefit to the merchant.*

For example, the operators plan to hold a sales promotion and will be recommended routes for this promotion. These routes not only have a high preference degree for this promotion but also bring pedestrian flow to increase potential benefits.

### 5.2. Learning Route Representations

The workflow to recommend routes for events is presented as flows: We first learn routes’ latent vector representations from context routes in the trajectories. Then, we capture events’ flow-aware preference for routes and learn events’ latent vector representations. At last, we recommend top-*N* routes for an event by the inner product of the latent factor vectors of an event and routes.

**Figure 5:**
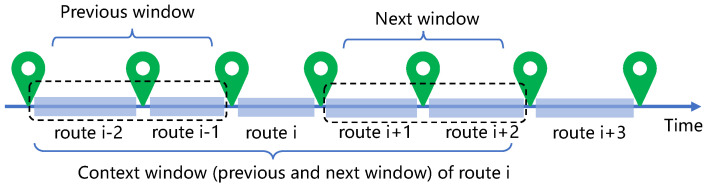
An instance of the context window of a trajectory. The window includes 4 routes visited before and after the *i*th route.

Based on the Skip-Gram model [8], we propose an embedding model to learn route representations, which captures routes’ contextual information from trajectory sequences. We learn the representations of context routes from $e_{i+k}$ to $e_{i-k}$ given a target route $e_{i}$. Here, *k* is set to control the window size of context, as shown in Figure 5. In our scenario, the collected route corpus is fed into the Skip-gram model. Compared with the word corpus, each route and each travel trajectory correspond to a word and a sentence, respectively. We aggregate all travel trajectories to construct a route corpus. We learn the route embeddings by maximizing the function below:

(13) $$\begin{array}{cc} \; & L_{m}=\sum_{S_{u}\in S}\frac{1}{|S_{u}|}\sum_{e_{i}\in S_{u}}\sum_{-k\leq z\leq k,z\neq 0}\log P\left(e_{i+z}|e_{i}\right) \end{array}$$

where *S* is a set containing all travel trajectories. $L_{m}$ aims to maximize the context route’s conditional occurrence likelihood for all travel trajectories.

In addition, we formulate the probability $P(e_{i+z}|e_{i})$ using a softmax function (The bold type in this paper refers to a vector.):

(14) $$\begin{array}{cc} \; & P\left(e_{i+z}|e_{i}\right)=\frac{\exp(v_{i+z}\cdot v_{i})}{\sum_{e_{x}\in E}\exp\left(v_{x}\cdot v_{i}\right)} \end{array}$$

where *D* refers to the dimension of the latent space; $v_{i}\in R^{D\times 1}$ and $v_{i+z}\in R^{D\times 1}$ refer to the latent representations of the target route $e_{i}$ and one of its context routes $e_{i+z}$, respectively. It is hard to directly optimize Equation (14) as the size of set *E* is extremely large. In this work, a negative sampling [8] method is utilized to promote optimization efficiency. For each route $e_{i}\in E$, a set of *K* routes that not occur in $e_{i}$’s context window are sampled. We reformulate the objective function as a new form that is easier to minimize,

(15) $$\begin{array}{cc} L_{m}= & -\sum_{S_{u}\in S}\frac{1}{|S_{u}|}\sum_{e_{i}\in S_{u}}\sum_{-k\leq z\leq k,c\neq 0}(\log\sigma \left(v_{i+z}\cdot v_{i}\right)+\sum_{k=1}^{K}E_{k}\log\sigma \left(-v_{k}\cdot v_{i}\right) \end{array}$$

where σ(·) refers to the commonly used sigmoid function. The noise distribution $P_{n}\left(r\right)$ is applied to sample the *K* negative routes, this distribution is the unigram distribution raised to the 3/4rd power [8]. E(·) means calculating the expected value for all negative samples used. We utilize the backpropagation algorithm to fit the Skip-gram model.

### 5.3. Flow-Aware Recommendation Model

In the above subsection, routes’ latent representations are learned by utilizing the context patterns in a global view, so the various flow characteristics on the routes are not considered. In order to address this problem, we design a flow-aware preference learning model for top-*N* route recommendations as events’ route selection. It is seen that the events are usually taken on the routes, so the flow size on the route is crucial for events. We define $f_{a,e}$ to denote the pedestrian flow size when an event *a* is taken on the route *e*. Note that the larger flow size *f* on the route *e*, the more confident that event *a* fit for the route *e*. Thus, the events’ preference rankings of the routes can be obtained. For example, $f_{a,e}>f_{a,e^{\prime }}$ indicates the route *e* ranked higher than the route $e^{\prime }$ for a given event *a*. In this paper, $f_{a,e}=0$ shows *a* is not fit for *e*. It can also refer to the fact that *a* has not been taken on *e*.

In order to design a flow-aware preference learning model for top-*N* route recommendation, with the help of pair-wise ranking loss, we utilize the Weighted Approximately Ranked Pairwise (WARP) loss [39] to learn events’ latent representations. By leveraging the precision@*N* measure, the WARP loss measures the pair-wise violations relying upon routes’ positions in the ranking list. For each event *a*, its arranged route set and un-arranged route set are denoted as $Z_{a}^{+}$ and $Z_{a}^{-}$, respectively. We define the WARP loss as follows,

(16) $$\begin{array}{cc} \; & L_{warp}=\sum_{a\in A}\sum_{e\in Z_{a}^{+}}L\left[rank\left({\hat{\omega }}_{a,e}\right)\right] \end{array}$$

where $rank({\hat{\omega }}_{a,e})$ is the rank of an arranged route $e\in Z_{a}^{+}$ in *a*’s flow-aware ranking list of routes. $rank({\hat{\omega }}_{a,e})$ is estimated by $\sum_{e^{\prime }\in Z_{a}^{-}}I\left({\hat{\omega }}_{a,e^{\prime }}\geq {\hat{\omega }}_{a,e}\right)$, and I(·) is an indicator function. To optimize the WARP loss function, we supplant the discrete indicator function by a continuous margin function [33]: $max(0,1-{\hat{\omega }}_{a,e}+{\hat{\omega }}_{a,e^{\prime }})$. L(·) is applied to convert a ranking order to a loss value, and $L\left(s\right)=\sum_{i=1}^{s}\frac{1}{i}$. ${\hat{\omega }}_{a,e}$ represents an event *a*’s preference for a route *e*, and it can be predicted by our factorization model: ${\hat{\omega }}_{a,e}=a^{⊤}v_{e}$, where $a\in R^{D\times 1}$ is the latent vector of an event *a*, and $v_{e}$ refers to *e*’s latent vector that obtained by the Skip-gram model in the Section 5.2. To accommodate the Skip-gram model, we set the dimension of events’ latent representations is the same as routes’ latent representations.

For most events, the number of routes un-arranged is substantially more than the arranged ones. We need to approximate the rank function efficiently. For each event *a*, we sample an un-arranged route $e^{\prime }$ randomly when an arranged route *e* is given, until the sampled routes do not conform with the margin function. ${\hat{\omega }}_{a,e}$ is estimated by $\left\lfloor \frac{|Z_{a}^{-}|-1}{N}\right\rfloor$, where ⌊·⌋ is floor function to obtain an integer, and N is the quantity of sampling times, |·| is the set’s cardinality.

We need to catch events’ flow-aware preference for routes, so we consider the flow size on the routes. Thus, we propose the F-WARP loss to enhance the WARP loss for more reasonable recommendations. For each pair of positive and negative routes $\left(e,e^{\prime }\right)$, we consider adding a weight $\theta_{e,e^{\prime }}$, which is defined as: $\theta_{e,e^{\prime }}=1+\gamma \cdot \left(f_{a,e}-f_{a,e^{\prime }}\right)$, where γ is to control the difference of increasement. If the difference is larger, this pair of routes does not conform to the margin function, so this pair of routes contribute much to the total loss. When we take the weight of the route pair into account, we reformulate the loss function as follows:

(17) $$\begin{array}{cc} L_{f-warp} & =\sum_{a\in A}\sum_{e\in Z_{a}^{+}}L\left[\sum_{e\in Z_{a}^{-}\cup \left\{Z_{a}^{+}\backslash e\right\}}max\left(0,\theta_{e,e^{\prime }}\cdot \left(1-{\hat{\omega }}_{a,e}+{\hat{\omega }}_{a,e^{\prime }}\right)\right)\right]+\lambda \sum_{a\in A}{\left\|a\right\|}_{F}^{2} \end{array}$$

where λ manages the degree of regularization and aims to avoid over-fitting. In the negative sampling routes process. It is not essential to bind the negative routes to un-arranged routes; the arranged routes of the event with a small flow size can likewise be sampled as negative cases.

We employ stochastic gradient descent (SGD) to learn the latent factors of events. For each arranged route, we sample a negative route to update the event’s latent factors in each iteration. The gradient of $L_{f-warp}$ about the *k*th latent factor of a is calculated as follows:

(18) $$\begin{array}{cc} \; & \frac{\partial L_{f-warp}}{\partial a_{k}}\;=\;L\left(\left\lfloor \frac{|E|-2}{N}\right\rfloor \right)\theta_{e,e^{\prime }}\left(v_{e^{\prime },k}\;-\;v_{e,k}\right)+2\lambda a_{k} \end{array}$$

The update rule for the latent factor is:

(19) $$\begin{array}{cc} \; & a_{k}\leftarrow a_{k}-\eta \frac{\partial L_{f-warp}}{\partial a_{k}} \end{array}$$

where η is the learning rate.

After the latent representations of events have been learned, we evaluate the suitability of an event *a* for a route *e* by computing the inner product of a and $v_{e}$. We recommend the routes for an event by ordering the candidate routes in a descending sort of the predicted scores, and then we select top-ranked *N* routes for the recommendation. We define the flow-aware route recommendation for business events based on the Skip-gram model as SG-FWARP.

## 6. Experiments

This section analyzes the pedestrian flow before comparing the performance of the ABMF and SG-FWARP with existing approaches.

### 6.1. The Experimental Setup

(1) Datasets. In the experiment, we describe three datasets as follows:

- The first is a surveillance video dataset of the New York Grand Central Station [40], which records people coming and passing by.
- The second one is a simulation dataset, which simulates pedestrian flow under different business event sets by using the social force model [41]. In the simulation dataset, the number of locations ranges from 10–60, and there are 8 types of business events, such as promotions, exhibitions of cloths, games and so on, each type of business event has a preference to attract visitors.
- The third one is Foursquare [2,42], a publicly crowdsourced large-scale check-in LBSNs dataset is free available. Foursquare of New York (NYC) has 1,385,223 check-ins, whereas Foursquare of Tokyo (TKY) has 573,703 check-ins. We obtain the check-in flows and identify the business events using the dataset’s visit counts for the routes.

(2) Experimental Settings. We choose 80% of the datasets to train the ABMF model, and the remaining 20% to test. We use cross-validation in training SG-FWARP. $\lambda_{m}$ is set to 0.0001, whereas $\lambda_{x},\lambda_{y},\lambda_{h}$ and $\lambda_{f}$ are all set to 0.01. *z* is assigned to different values based on the simulation data’s scale. η is set as 0.001 first, and we decrease its value when iterations are sufficient. λ is set to 0.01. We recommend top-10 routes for a business event. We select the regularization coefficient by experimental value. The process is as follows: we first determine the learning rate; then we determine the order of magnitude of $\lambda_{m}$ (such as 0.0001 or 0.01, etc). Finally, we fine-tune it further to obtain a proper value of a regularization coefficient.

### 6.2. Metrics

- The ABMF model is evaluated according to matrix similarity and root-mean-square error (RMSE), which are defined as:
  (20) $$\begin{array}{cc} \; & sim\left(M,N\right)\;=\;\frac{\sum_{i}\sum_{j}\left(m_{ij}\;-\;\bar{M}\right)\left(n_{ij}\;-\;\bar{N}\right)}{\sqrt{\left(\sum_{i}\sum_{j}{\left(m_{ij}\;-\;\bar{M}\right)}^{2}\right)\left(\sum_{i}\sum_{j}{\left(n_{ij}\;-\;\bar{N}\right)}^{2}\right)}} \end{array}$$
  (21) $$\begin{array}{cc} \; & RMSE=\sqrt{\frac{1}{n}\sum_{i=1}^{n}\sum_{j=1}^{n}\left(m_{ij}-n_{ij}\right){)}^{2}} \end{array}$$
  where sim(M,N) represents the similarity degree between *M* and *N* adjusted to 0−1. Both *M* and *N* are *n*-order matrices that represent the ground truth and predicted pedestrian flow matrix, respectively. The average values of matrix *M* and matrix *N* are represented by $\bar{M}$ and $\bar{N}$, respectively.
- To evaluate the performance of route recommendation for events, we use precision@N, which refers to the ratio of the successfully predicted routes to the top-N recommendations. We use Mean Reciprocal Rank (MRR) as another metric. This ranking metric measures the recommendation accuracy by finding out how far the first successfully predicted route is from the top of the recommendation list. MRR is defined as follows:
  (22) $$\begin{array}{cc} \; & MRR=\frac{1}{A}\sum_{i=1}^{\left|A\right|}\frac{1}{r_{i}} \end{array}$$
  where |A| is the size of the event set. For the *i*th event, $r_{i}$ refers to the ranking position of the first route in the recommendation list in the ground-truth result. Note that all experiments are conducted 10 times for latent factor models, and we report the averaged results.

### 6.3. Baseline methods

- The ABMF model is used to predict pedestrian flow based on a set of business events. We compare the prediction results of the ABMF model with the other three prediction methods. The baseline algorithms include vSVR [21], PMF [37,43], and MOHER [18] (1) vSVR is an application of SVM (Support Vector Machine) to regression problems, which can be applied to predict the pedestrian flow density, and then covered to the number of individuals. (2) PMF is an effective method that is frequently used as a baseline in current work [44], which factors the pedestrian flow matrix into two feature matrices. (3) MOHER is to predict the potential crowd flow in a certain mode, which uses the LTSM module to predict the sequential flow. However, the baseline algorithms cannot reflect the diverse influence of business events, because they fuse the influence of business events into the pedestrian flow.
- We compare SG-FWARP with three top-N recommendation methods. (1) The first is WRMF [45], which is the weighted regularized matrix factorization model designed to handle implicit feedback data (i.e., an event taken on a route or not) for top-N recommendations. This method is used as a baseline in the latest work [46]. (2) The second one is WARP-MF [47]. This is a pairwise ranking method that utilizes matrix factorization to minimize the basic WARP loss. The latent factors of events and routes are learned by randomly sampling the positive and negative route pairs. (3) The third one is similarity pairwise ranking matrix factorization (SPRMF) [48]. This method uses a new penalty to eliminate the differences in the scores between popular and personalized items based on their similarity.

### 6.4. Analysis of Pedestrian Flow

**Figure 6:**
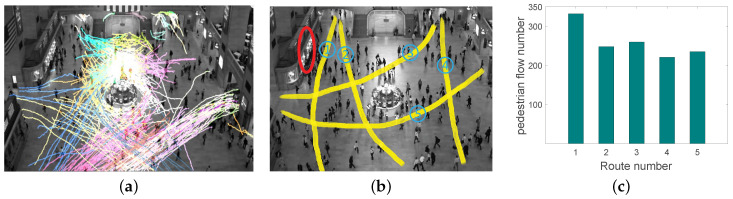
The statistics in New York Grand Central Station. (**a**) Trajectory clustering; (**b**) The extracted trajectories; (**c**) The pedestrian flow number.

A 5-min video collected from New York Grand Central Station is used to analyze the pedestrian flows. We use KLT keypoint tracker [49] to extract trajectories. Figure 6a shows the clustered main coarse-grained trajectories. Then, the extracted trajectories are shown in Figure 6b. A red ellipse represents the attractive region. The pedestrians congregate around the attractive region, indicating that something draws pedestrians to visit. As shown in Figure 6c, the horizontal axis represents the indexes of the clustered routes extracted from the video in Figure 6b. The vertical axis reflects the number of pedestrian flows on each route during the video. According to the statistical results, the number of pedestrian flow on route 1 is higher than that of other routes, indicating that the attractive region draws in the pedestrians for route 1. As a result, it makes sense to introduce the attractive crowd index for each property in the ABMF model.

### 6.5. Experimental Results

We present the experimental results of the two problems separately.

#### 6.5.1. Experimental Results for the ABMF

**Figure 7:**
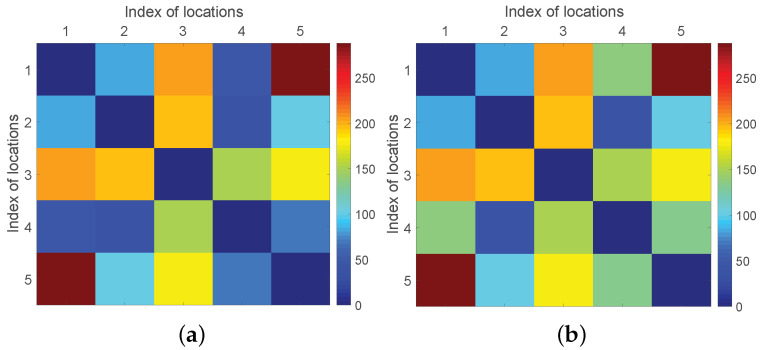
Heat map of pedestrian flow matrix: (**a**) The original matrix; (**b**) The changed matrix.

Based on the simulation pedestrian dataset with business events, the pedestrian flow matrix without/with business events are mapped on the heat map. Figure 7a shows the original pedestrian flow matrix with five locations, and Figure 7b shows the pedestrian flow matrix of five locations when arranging two business events on $e_{14}$ and $e_{54}$, respectively. It is obvious that the pedestrian flow values on route $e_{14}$ and $e_{54}$ in Figure 7b are larger than the corresponding values in Figure 7a, indicating that the scheduled business events draw additional pedestrian flow for the associated routes.

**Figure 8:**
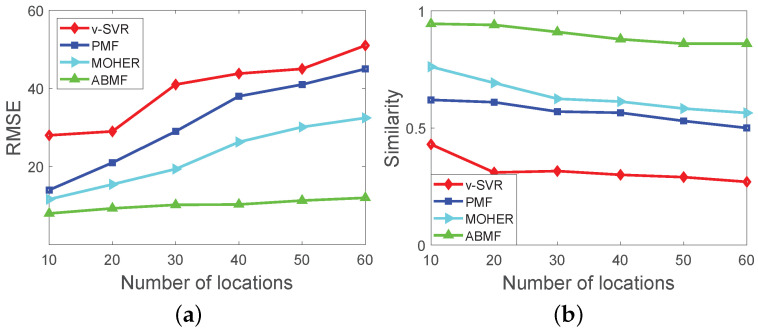
Performance comparison with different scale of simulation dataset: (**a**) Performance on RMSE; (**b**) Performance on Similarity.

**Figure 9:**
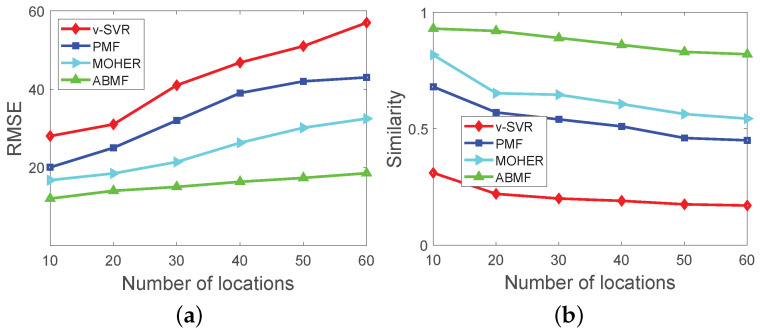
Performance comparison with different scale of Foursquare(NYC) dataset: (**a**) Performance on RMSE; (**b**) Performance on Similarity.

Figure 8 depicts the performance of various algorithms with various scales of simulation datasets. In Figure 8a, the number of locations corresponds to the scale of the simulation dataset, and each business event set has the same categories under all the simulation datasets. The dimension of latent features is set differently with the different scales of the datasets. The number of locations in the dataset ranges from 10 to 60, and the corresponding dimension is set from 5 to 30. Overall, the RMSE of the ABMF is lower than that of the other three methods. The smaller RMSE is, the more accurate the algorithm is. The reason is that ABMF considers the ability of categories to attract pedestrian flow and refines the source of the flow of a route. The performance of PMF is better than that of vSVR because PMF considers potential factors in matrix factorization. In Figure 8b, the similarity between the predicted matrix by the ABMF and the ground truth matrix is higher than that of the other three algorithms overall. Regarding the metric of similarity, its range is 0 to 1, and the greater the similarity, the closer the predicted result is to the ground truth. As the ABMF takes into account the entire flow sources of a route with a business event, the ABMF is utilized for pedestrian flow prediction with the diversity of business events set. The result shows that the ABMF outperforms vSVR, PMF, and MOHER, because the three methods fuse the influence of business in the model, while ABMF models the attraction of business events to the pedestrian flow in the design. MOHER performs better than vSVR and PMF because MOHER uses LSTM to mix different flow modes and then predict the pedestrian flow. The performance of MOHER is not as good as ABMF, because MOHER is more suitable for the mix of different transportation modes, and only predicting pedestrian flow can not play a full role. In Figure 9, ABMF performs well on large-scale real datasets, and its performance is superior to vSVR, PMF, and MOHER. The analysis in Figure 9 with the Foursquare dataset is consistent with that in Figure 8.

**Figure 10:**
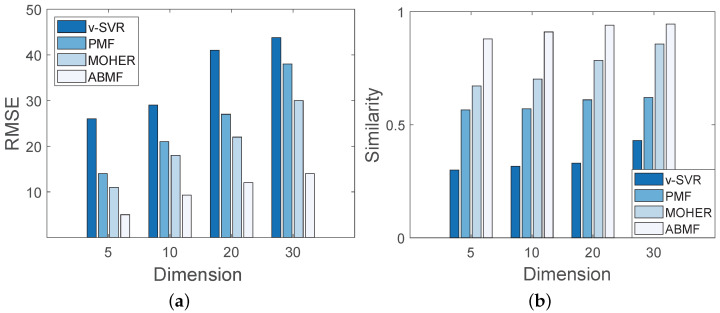
Performance with different dimensions on simulation dataset: (**a**) Performance on RMSE; (**b**) Performance on Similarity.

**Figure 11:**
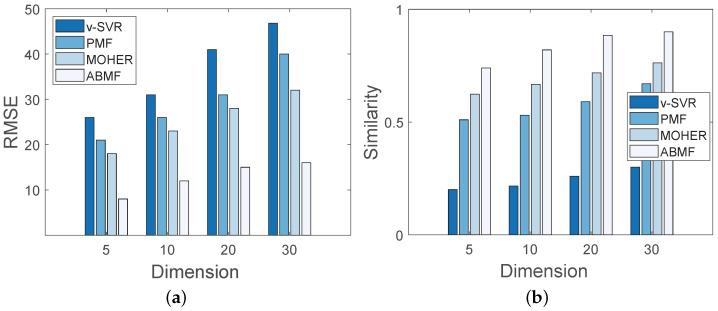
Performance with different dimensions on Foursquare (NYC) dataset: (**a**) Performance on RMSE; (**b**) Performance on Similarity.

In this experiment, the simulation data is associated with 20 locations and 8 kinds of business events, and the dimension of location latent factor *z* is set to different values in order to evaluate the metrics of similarity and RMSE. Figure 10 displays the performance of various algorithms with varying dimensions of the latent factor. As illustrated in Figure 10b, the similarity of both four methods rises with the dimension increasing. In Figure 10a, the RMSE of the ABMF is lower than that of the other three methods with the dimension increasing, indicating that a larger dimension model is more accurate in predicting pedestrian flow, and the ABMF model performs the best of the four algorithms. When the dimension is set to 30, the similarity of the ABMF remains close to 1, indicating that the ABMF performs well in terms of accuracy and stability. The analysis in Figure 11 under the Foursquare dataset is similar to that in Figure 10. Figure 11 shows that when the dimension increases, the performance of ABMF in terms of RMSE and similarity changes little, indicating the solid performance of the ABMF model. The increase in the potential feature dimension plays a promotive role in the performance of ABMF.

**Figure 12:**
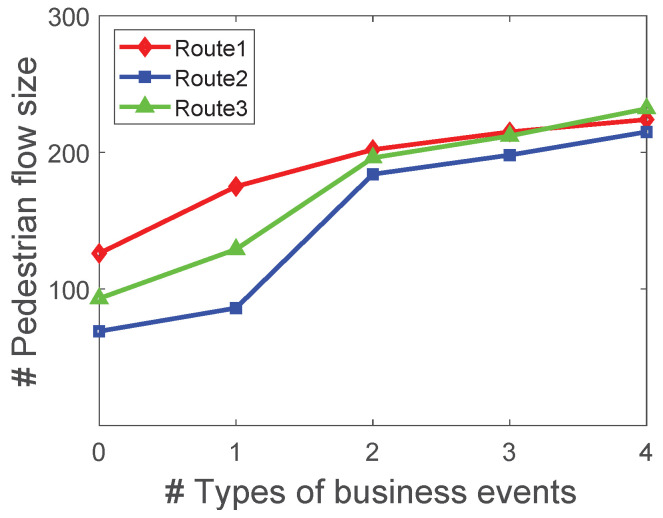
The impact of diversity events on the route in the simulation dataset.

Figure 12 displays the influence of business event diversity on the routes in the simulation dataset. There are four different types of business events in the business events set; each business event is associated with a type. We select three routes from the simulation dataset, and we place 0,1,2,3 or 4 business events on each route to obtain the changed pedestrian flow matrix, respectively. Each time, the business events are placed on the three routes simultaneously. The results show that the pedestrian flow size of each route rises as the type of business events increases.

#### 6.5.2. Experimental Results for SG-FWARP

We first analyze how the context window size impacts the performance of SG-FWARP in Table 2. The dimensionality of latent factor vectors *D* is set as 300 in the experiments. The size of the context window varies from 1 to 6. From Table 2, we observe that precision and MRR increase first with the increasing context window size, then decrease after arriving at a larger value at the window size of 3 or 4. The reason is as follows: a larger window size represents the target route’s context and could be more comprehensively modeled. Compared with the large-scale text corpus where the Skip-gram is typically used, the route corpus we used is sparse, so a small context window size is proper to model the route’s context influence in our experiments.

**Figure 13:**
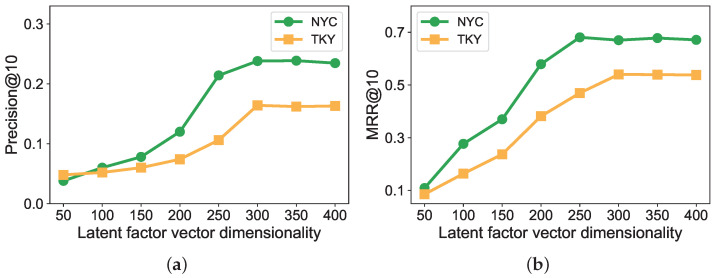
Performance of different latent factor vector dimensionality (top-10 recommendations): (**a**) Precision; (**b**) MRR.

Next, we report the impact of dimensionality on the latent vectors in Figure 13. In the SG-FWARP method, we set the dimensionality from 50 to 400 with 50 as the increment. Figure 13 shows that a higher latent vector dimensionality brings higher precision and MRR. This trend reflects that higher latent vector dimensionality helps to capture more latent factors and more accurately represent routes and events. However, we find that precision and MRR are stable after achieving a certain threshold (e.g., 300). As a consequence, an optimal latent vector dimensionality can be obtained from empirical value, so we can obtain a high recommendation quality with proper computational overheads. In the following comparison experiment, we set 300 as the default dimensionality of a latent vector.

In the comparison experiment, we compare the performance of our proposed method SG-FWARP with three baseline methods. By using cross-validation, for the methods WRMF, WARP-MF, and SPRMF, we set the dimension of latent factor vector, γ, and regularization coefficient λ as 200, 1, and 0.01, respectively. For SG-FWARP, the latent factor vector dimensionality, γ, and regularization coefficient are set to 300, 1, and 0.01, respectively. In addition, the optimal context window size is set for different datasets.

Table 3 shows the experimental results on two datasets when evaluating top-10 route recommendations as events’ route recommendations. We can see that the SG-FWARP model outperforms WARP-MF, WRMF, and SPRMF, because SG-FWARP captures the semantic information of sequence routes. The main difference is that semantic information (i.e., categories of routes) is considered to learn the latent preferences, thus improving the performance. The other three algorithms WARP-MF, WRMF, and SPRMF fuse the flow factor in the matrix factorization, while SG-FWARP model introduces the flow factor into the WARP loss to improve the reasonability in flow-aware route recommendation for business events.

Overall, SG-FWARP is superior to the three baseline methods by two main design mechanisms. First, we apply the Skip-gram model to learn the routes’ latent representations to capture the context (before and after the target route) of routes. Second, based on the learned route latent representations, the F-warp loss function combines the flow size of the routes with a pairwise ranking algorithm to learn users’ latent representations for flow-aware route recommendation.

### 6.6. Discussion

Overall, the problem of route recommendation for business events happens before the pedestrian flow prediction. Route recommendation for business events is to assist operators in making decisions when the operators have no idea where to place the business event. We explained the experimental results in the above subsections in detail. We first verify the performance of ABMF in predicting pedestrian flow, then we show the experimental results to demonstrate the feasibility of recommending routes for business events. However, suppose the operators apply SG-FWARP every time they make a decision. In this case, the problem of pedestrian flow prediction with business events and the problem of route recommendation for business events will affect each other in the long term. Below, we conclude our paper and present future work.

## 7. Conclusions

In this paper, we first present the ABMF model to predict the pedestrian flow with business events, which is essential because pedestrian flow can bring potential profits to operators; the highlight of the ABMF model is that it introduces the attraction index of different categories to pedestrians in matrix factorization. Then, we learn route representations based on the Skip-gram model, and we improve the basic pair-wise ranking loss to a flow-aware-based method by considering the flow factors, which aims to learn events’ latent representations for route recommendation. To the best of our knowledge, we are the first to predict pedestrian flow by considering business events. We evaluate the performance of the ABMF and SG-FWARP; both are superior to the baseline methods, which confirms the ABMF and SG-FWARP models are better and more applicable to pedestrian flow prediction with business events and route recommendation for business events. The limit of this paper is that we did not consider the interaction effect between route recommendation for the events and pedestrian flow prediction; the two problems can affect each other in the long run. In the future, we will consider the joint optimization of the two problems and find the optimal solutions under the interaction effect by utilizing Markov Process. Furthermore, we will also consider the time-aware factors to improve the ABMF and SG-FWARP models.

## Figures and Tables

**Table 1:** Frequently-used notations.

Notation	Interpretation
V/E	the set of locations/routes, $v_{i}\in V,\left|V\right|=n$, $e_{ij}\in E$
*A*	the business events set, $a_{i}\in A$
$A^{\prime }$	the current business events set
MA	the set of pedestrian flow history data
$M^{i}$	the *i*th pedestrian flow matrix
$A^{i}$	the *i*th the business events set, $A^{i}\subseteq A$
$R^{i}$	the position matrix of $A^{i}$
*C*	the set of categories, $c_{i}\in C$
*X*	the latent location feature matrix
*H*	the latent business event feature matrix
*Y*	latent factor feature matrix of location
*F*	latent factor feature matrix of business event
$f(a_{i})$	the function to map $a_{i}$ to its category index
$\lambda_{x},\lambda_{y},\lambda_{h},\lambda_{f}$	the regularization parameters of *X*, *Y*, *H* and *F*
$\lambda_{m}$	the reciprocal of the variance
η	the learning rate
*s*	the predicted pedestrian flow matrix index
*z*	the latent feature dimension
$S_{i}$	the *i*th trajectory, $S_{i}\in S$
v/a	the vector of route/event
$f_{a,e}$	the flow size with event *a* on the route *e*
$L_{m}$	loss function of learning route representations
$L_{warp}$	loss function of flow-aware WARP loss

**Table 2:** Performance of different context window size on SG-FWARP (top-10 recommendations).

Dataset	Metric	Context Window Size					
		1	2	3	4	5	6
Foursquare (NYC)	Precision@10	0.2024	0.2342	0.2548	0.2746	0.2383	0.2262
	MRR@10	0.5656	0.6198	0.6841	0.7071	0.6108	0.5026
Foursquare (TKY)	Precision@10	0.0983	0.1424	0.1663	0.1569	0.1322	0.1172
	MRR@10	0.4318	0.4655	0.5645	0.5142	0.4731	0.4128

**Table 3:** Performance comparison on top-10 route recommendation.

Dataset	Algorithm	Precision	MRR
Foursquare NYC	WRMF	0.1392	0.3712
	WARP-MF	0.1253	0.3517
	SPRMF	0.1927	0.5461
	SG-FWARP	0.2436	0.6814
Foursquare TKY	WRMF	0.0912	0.2310
	WARP-MF	0.0836	0.2150
	SPRMF	0.1261	0.3952
	SG-FWARP	0.1632	0.5147

## Data Availability

The data presented in this study are available on request from the first author.
